# The health and economic burden of haemophilia in Belgium: a rare, expensive and challenging disease

**DOI:** 10.1186/1750-1172-9-39

**Published:** 2014-03-21

**Authors:** Séverine Henrard, Brecht Devleesschauwer, Philippe Beutels, Michael Callens, Frank De Smet, Cedric Hermans, Niko Speybroeck

**Affiliations:** 1 Institute of Health and Society (IRSS), Université catholique de Louvain, Brussels, Belgium; 2 Haemostasis and Thrombosis Unit, Division of Adult Haematology, Cliniques universitaires Saint-Luc, Brussels, Belgium; 3 Department of Virology, Parasitology and Immunology, Faculty of Veterinary Medicine, Ghent University, Salisburylaan 133, 9820 Merelbeke, Belgium; 4 Centre for Health Economic Research and Modeling Infectious Diseases (CHERMID), Vaccine and Infectious Disease Institute (Vaxinfectio), University of Antwerp, Wilrijk, Belgium; 5 National Alliance of Christian Sickness Funds, Brussels, Belgium; 6 Department of Public Health and Primary Care, Occupational, Environmental & Insurance Medicine, Katholieke Universiteit Leuven, Leuven, Belgium

**Keywords:** Burden, Costs, Haemophilia, Disability-adjusted life year

## Abstract

**Background:**

Haemophilia is a rare hereditary haemorrhagic disease that requires regular intravenous injections of clotting factor (CF) concentrates. This study sought to estimate the health and economic burden of haemophilia in Belgium. This is the first study of its type to be conducted, and reflects the Belgian authorities’ growing interest for haemophilia as part of their priority planning for rare and chronic diseases.

**Methods:**

A probabilistic model was developed in order to estimate the lifetime haemophilia burden for the 2011 birth-year Belgian cohort. The health burden was initially expressed in terms of disability-adjusted life years (DALYs), the number of healthy life years lost due to living with disability and dying prematurely. An incidence perspective was used in line with World Health Organization recommendations. The economic burden calculated from direct and indirect haemophilia-related costs was expressed in euros. Data were drawn from the literature if none were available from federal institutions or health insurance. Disability weights for DALY calculation were derived using generic quality-of-life tools such as SF-6D from the SF-36 (36-item Short-Form Health Survey; for adults) and KINDL (generic quality-of-life instrument; for children) compared to population norms. Analyses were stratified according to haemophilia type and severity.

**Results:**

In Belgium, haemophilia resulted in 145 undiscounted and unweighted DALYs in total (95% credible interval [CrI] = 90-222), which represents an average of 11 DALYs per incident case with haemophilia (95% CrI = 8-15) during his life, varying according to haemophilia severity (17 DALYs for severe haemophilia, 12 DALYs for moderate, and 4 DALYs for mild). Mean total lifetime costs reached €7.8 million per people with haemophilia, 94.3% being direct costs and 5.7% indirect costs. Clotting factors accounted for 82.5% of direct costs.

**Conclusions:**

Haemophilia represents both an economic and health burden, especially regarding individual health on an individual patient level. Initiatives to counteract this burden should be clearly identified and given full support, as this burden is likely to increase in the future, especially from an economic perspective. Our study may also contribute towards a better global evaluation of haemophilia in the future.

## Introduction

Haemophilia A and B are rare X-linked haemorrhagic disorders, caused by a lack of circulating clotting factor VIII (FVIII) or factor IX (FIX), respectively, in blood. Men are affected by the disease, while women are carriers. Haemophilia severity, which depends on circulating clotting factor levels in the blood, is defined as follows: severe (<1 IU/dL), moderate (1–5 IU/dL) or mild (6–40 IU/dL) [1]. If untreated, the bleeding tendency in haemophiliacs may induce spontaneous bleedings leading to premature death. However, treatments exist today that allow many haemophiliacs to benefit from a close-to-normal life expectancy, with the exception of a certain number of severe haemophiliacs [2].

Before the 1990s, standard treatment for people with haemophilia (PWH) consisted of infusions of human plasma-derived clotting factor products. In the early 1980s, however, the emerging human immunodeficiency virus (HIV) and hepatitis C epidemics had a devastating impact on PWHs treated with this type of human plasma product. The introduction of heat-treated concentrates in 1985 diminished the risk of HIV and hepatitis C infections [3], while the first recombinant clotting factor products (in 1992 for FVIII and 1997 for FIX) resulted in the eradication of haematogenous HIV and hepatitis C infections. Another major complication of haemophilia includes recurrent spontaneous bleedings in joints and muscles, which can lead to haemophilic arthropathies in the long term, with severe impairments, activity limitation, and thus productivity loss [4,5]. The development of inhibitors to the clotting factor, resulting in partial or complete lack of FVIII or FIX efficiency, is another issue [4]. All these complications can seriously impair the patients’ quality of life, with potential implications for employment, productivity, and patient psychological well-being [6,7]. In the past, untreated PWHs, and particularly those with severe disease, tended to die earlier than the general male population, with a life expectancy below 30 years in 1960, and then below 60 years in 1980. Since the 1990s, the development of virus inactivation techniques for preparing plasma-derived concentrates, as well as recombinant concentrates, have improved PWH life expectancy, so much so that it now approaches male life expectancy in the general population of high- and middle-income countries [8]. As a consequence, haemophilia prevalence has been increasing over recent years.

While appropriate haemophilia management is essential to reduce, or better prevent long-term implications, it considerably increases costs for PWH and the rest of society [4]. One appropriate management option consists of prophylactic treatment administration, given one to three times a week.

To our knowledge, the haemophilia health and economic burden in Belgium has not yet been assessed. This study pursued two principal objectives: 1) to estimate the health burden of haemophilia in Belgium by using the disability-adjusted life year (DALY) approach [9]; 2) to assess the economic burden by calculating both direct and indirect costs. Our study was in line with the growing interest of authorities in rare and chronic diseases, such as haemophilia, with respect to national management strategies in Europe [10].

## Methods

### Birth cohort

We estimated the lifetime burden of haemophilia for the 2011 birth-year Belgian cohort. To this end, we simulated the number of new-born males with haemophilia in 2011, taking into account the number of male births in 2011 (67,149) along with a haemophilia incidence ranging uniformly from 1/5500 to 1/4500 new-born males [11-14]. The proportions of severe, moderate and mild haemophilia A and B cases were modelled using Belgian Haemophilia Association data (Table 1). For severe haemophiliacs, we modelled the excess mortality compared to the general 2011 Belgian mortality rates, using the haemophilia standardized mortality rates provided by Plug *et al.* (2006; Table 1) [15]. For moderate and mild haemophiliacs, we assumed that there was no haemophilia-related mortality [16]. To allow for international comparisons, we modelled the life expectancy of surviving patients using the Coale-Demeny West model life table, displaying a male life expectancy at birth of 80 years [17]. To account for parameter uncertainty, we simulated 10,000 birth cohorts, using different randomly drawn values of the various input distributions. All simulations and calculations were conducted using the R Software version 2.15.2 [18].

**Table 1:** Distributions used to model the 2011 birth-year Belgian cohort of haemophilia patients

**Parameter**	**Distribution**	**Reference**
Incidence	Uniform (min = 1/5500, max = 1/4500)	Rosendaal and Briet [11]
Number of haemophilia A, haemophilia B cases	Dirichlet ({941, 212})	Belgian Haemophilia Association (year 2011)
Number of severe, moderate, mild haemophilia A cases	Dirichlet ({440.4, 180.7, 319.9})	Belgian Haemophilia Association (years 2006–2011)*
Number of severe, moderate, mild haemophilia B cases	Dirichlet({63.6, 63.6, 84.8})	Belgian Haemophilia Association (years 2006–2011)*
Standardized mortality rate	Beta-PERT (min = 0.8, mode = 1.4, max = 2.4)	Plug *et al.*[15]

*The estimated number of severe, moderate and mild haemophilia cases was calculated using the mean number of new cases with haemophilia between 2006 and 2011 from the Belgian Haemophilia Association data; Dirichlet: Dirichlet distribution; Beta-PERT: Beta-PERT distribution.

### Disease burden

Disability-adjusted life years (DALYs), which represent the number of healthy life years lost due to haemophilia, are calculated by adding the adjusted number of years lived with disability (YLDs) to the number of years of life lost due to premature mortality (YLLs). In their simplest form, the formulae for YLDs and YLLs are [19]:

$$\begin{array}{l} \text{YLD}\;=\;\text{number}\;\text{of}\;\text{incident}\;\text{cases}\;\times \;\text{duration}\;\text{of}\;\text{the}\\ \;\text{disease}\;\times \;\text{disability}\;\text{weight}\\ \text{YLL}\;=\;\text{number}\;\text{of}\;\text{deaths}\;\times \;\text{standard}\;\text{life}\;\text{expectancy}\;\text{at}\\ \;\text{age}\;\text{of}\;\text{death} \end{array}$$

Disability weights (DW) are a crucial factor in YLD calculation, as they allow for adjustment of the number of years lived with disability and for comparison with the number of life years lost due to premature mortality [20]. The haemophilia DW indicates to which extent haemophilia reduces the patient’s physical capacity, ranging from zero (full health) to one (worst possible health state). As DWs are dependent on social choices, several sets of DWs have been developed, such as the Global Burden of Disease (GBD) DWs [21] and the Dutch Severity Weights [22].

Since no haemophilia-specific DWs were available either in the Global Burden of Disease list nor the Dutch Disability Weights Group, data from a generic-based questionnaire for both adults and children were used. As no quality-of-life evaluation using generic assessments in haemophiliac children was available in Belgium, we took the DWs estimated from the mean KINDL score from KINDL questionnaire for children with haemophilia from the scientific literature [23] minus the mean KINDL score for boys from the normal population (population norms) for each specific age groups [24]. For adults, the DW estimation was based on the results of the SF-6D scores generated from the SF-36 quality-of-life questionnaire filled out by 71 PWHs in Belgium [25] minus the mean SF-6D score for males in the general population (population norms) and for each age groups [26]. The DWs chosen for both children and adults are shown in Table 2.

**Table 2:** Disability weights used for adults and children with haemophilia in the disability-adjusted-life-year calculations

	Disability weight*
Children	
6-7 years	0.018
8-12 years	0.064
13-17 years	0.039
Adults	
Severe	0.197
Moderate	0.151
Mild	0.054

*The most likely value for the disability weights was estimated using the KINDL questionnaire for children from the scientific literature [23] compared to KINDL population norms [24] and the SF-6D generated from the SF-36 quality-of-life questionnaire filled out by 71 PWHs in Belgium [25] compared to the SF-6D population norms [26].

Standard DALY calculation requires taking into account two other social preference criteria, namely age weighting and time discounting. Age weighting gives a higher significance to the period of 9 to 54 years of age, as these years are generally regarded as being of greater social value [27,28]. However, the limited empirical evidence of such an approach along with the resulting inequality between ages have attracted criticism. Time discounting reduces the impact of years lived in the future, generally at a rate of 3%. This strategy, which is in line with economic assessments, prevents policy-makers from investing in potential future eradication programs rather than control programs that are possible right now, yet perhaps less effective, which is known as the research paradox. Time discounting has also received criticism, mainly on the grounds of inequality as explained above. Given this controversy, we decided to report both undiscounted and unweighted DALYs as our base case scenario. However, to allow for comparison with other studies, we conducted scenario analyses, with DALYs calculated based on a 3% time discount rate, both with and without age weighting as well as 1.5% time discount rate without age weighting.

### Cost analysis

Cost estimations were performed using data from the National Alliance of Christian Mutualities (NACM) database, which is the largest Belgian sickness fund, covering approximately 42% of the population. All data extractions and analyses were conducted at the NACM medical management department under the supervision of a medical advisor. In Belgium, sickness insurance is legally compulsory. As no medical diagnosis was reported in the database, our cost calculations included all male patients who had at least one specific haemophilia medication injection in 2011, such as clotting factor VIII (ATC code B02BD02), clotting factor IX (ATC code B02BD04), nonacog alfa (BeneFIX) (ATC code B02BD09), factor VIII inhibitor bypassing activity (FEIBA) (ATC code B02BD03) and activated eptacog alfa (NovoSeven) (ATC code B02BD08), and who were still alive on December 31, 2011. For the patients selected between January 1, 2011 and December 31, 2011, health benefits and associated social security and patient costs were calculated and used as references in the cost calculations (see Additional files 1 and 2). In addition, the exact total 2011 costs of all above-mentioned specific haemophilia medications mentioned above was provided by the National Institute for Health Care and Disability Insurance. Using the NACM database, the following costs were estimated: number and mean cost of visits to general practitioner (GP), specialist, physiotherapist and dentist; number of days and average daily cost of hospitalisation hospital stay and day hospitalisation; average costs of other expenses. All these costs were considered direct costs. Calculated indirect costs included transport costs to and from the doctor’s office and hospital, as well as absence from work due to these appointments and hospitalisations. Transport costs were estimated based on a travel distance ranging uniformly from 0 to 200 km with a cost of €0.30 per km travelled; the daily cost of absence from work was assumed to equal the average gross daily income. The cost of absence from work due to invalidity or premature death was assessed for patients aged 20–64 by means of a friction-cost method [29]. Using this method, costs were only considered for the period required to replace a sick or deceased worker. In Belgium, however, precise data on the length of this so-called friction period was unavailable. This uncertainty was accounted for by modelling the friction as a uniform distribution ranging from 2 to 6 months [30]. The distributions used in the cost assessment model for the 2011 birth-year Belgian cohort of haemophilia patients are shown in Additional files 1 and 2. In the results section, we have estimated the lifetime costs for new incident haemophilia cases born in 2011 applying no time discounting and 3.5% time discounting [31].

### Sensitivity analyses

Probabilistic sensitivity analyses were conducted in order to assess the sensitivity of DALYs and cost estimates as affected by the uncertainty in the input parameters. Spearman’s rank-order correlations were employed to assess the association between the random deviates from the input parameters and the simulated DALY and cost estimates. The correlation coefficient for each input parameter was then taken as a measure of model sensitivity, with values higher than zero indicating positive associations and values lower than zero indicating negative associations.

## Results

### Disability-adjusted life years (DALY)

For the 2011 Belgian population, haemophilia was estimated to be the cause of a total of 145 (95% credible interval [95% CrI]: 90–222) healthy life years lost (DALYs), with 119 (95% CrI: 81–171) being due to years lived with disability (YLDs) and 26 (95% CrI: 0–64) years lost due to premature mortality (YLLs) (Table 3). DALYs were higher for haemophilia A (123 DALYs) than for haemophilia B patients (22 DALYs), given the higher haemophilia A’s incidence than haemophilia B. If we consider haemophilia severity, the mean number of DALYs per case was highest in severe haemophiliacs, with a mean of 17 DALYs per severe haemophiliac, whereas moderate haemophiliac cases had a mean of 12 DALYs, and mild haemophiliacs a mean of 4 DALYs (Table 3). The scenario analyses assessing the impact of alternative social preference functions on overall and individual lifetime haemophilia burden per haemophilia type for the 2011 birth-year Belgian cohort are provided in Table 4 in order to be able to compare the results obtained with these different scenarios with future studies. In brief, haemophilia resulted in 72 DALYs applying 1.5% time discounting and no age weighting, 40 DALYs applying 3% time discounting and no age weighting and 47 DALYs applying 3% time discounting and age weighting.

**Table 3:** Number of estimated new cases, overall and per case lifetime burden (Belgium, 2011)

	**Estimated number of new cases in 2011 (95% CrI)**	**DALY (95% CrI)**	**DALY per case (95% CrI)**
Haemophilia (total)	14 (7–22)	145 (90–222)	11 (8–15)
Severe	6 (3–10)	98 (43–175)	17 (13–20)
Moderate	3 (1–5)	29 (27–31)	12 (5–25)
Mild	5 (2–8)	18 (17–20)	4 (2–9)
Haemophilia A	11 (5–18)	123 (74–193)	11 (8–16)
Severe	5 (2–9)	86 (37–156)	17 (13–20)
Moderate	2 (1–4)	22 (20–23)	12 (5–28)
Mild	4 (2–7)	15 (14–17)	5 (2–10)
Haemophilia B	2 (1–5)	22 (12–42)	10 (5–18)
Severe	1 (0–2)	12 (2–32)	17 (13–20)
Moderate	1 (0–2)	7 (6–8)	16 (4–55)
Mild	1 (0–2)	3 (2–3)	4 (1–13)

95% CrI: 95% credible interval.

Number of estimated new haemophilia cases, overall lifetime haemophilia burden and lifetime haemophilia burden per case per haemophilia type and severity in Belgium in 2011.

**Table 4:** Scenario analyses on lifetime haemophilia burden for 2011 in Belgium

**Scenario**	**1.5% time discounting, no age weighting**		**3% time discounting, no age weighting**		**3% time discounting, age weighting**	
	**DALY**	**DALY per case**	**DALY**	**DALY per case**	**DALY**	**DALY per case**
	**(95% CrI)**	**(95% CrI)**	**(95% CrI)**	**(95% CrI)**	**(95% CrI)**	**(95% CrI)**
Haemophilia (total)	72 (46–108)	5 (4–8)	40 (26–58)	3 (2–4)	47 (31–69)	4 (3–5)
Severe	47 (21–82)	8 (7–9)	25 (11–43)	4 (4–5)	30 (14–51)	5 (5–5)
Moderate	15 (14–16)	6 (3–13)	8 (8–9)	3 (2–7)	10 (9–11)	4 (2–9)
Mild	10 (9–11)	2 (1–5)	6 (6–7)	1 (1–3)	7 (7–8)	2 (1–4)

95% CrI: 95% credible interval.

Results of the scenario analyses assessing the impact of alternative social preference functions on overall and individual lifetime haemophilia burden per haemophilia type for the 2011 birth-year Belgian cohort are shown.

**Figure 1:**
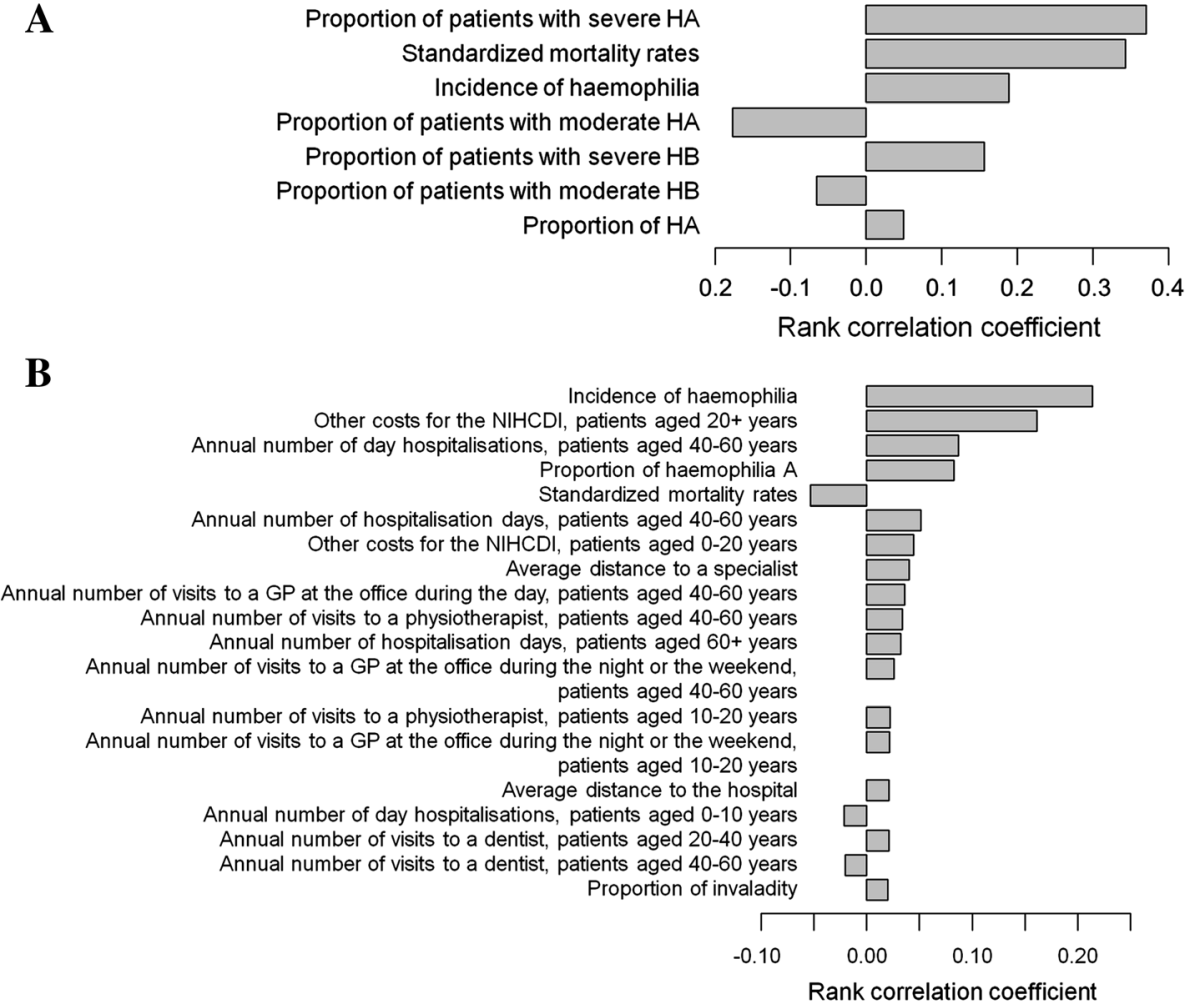
**Sensitivity analysis for DALY assessment (A) and cost estimation (B).** Only parameters with a correlation coefficient significantly different from zero are presented in the figure. HA: haemophilia A; HB: haemophilia B; GP: General practitioner.

Sensitivity analyses indicated results to be mostly influenced by the uncertainty in the proportion of severe haemophiliacs A, and then by the uncertainty in standardized mortality rates and incidence (Figure 1A).

### Cost estimation

The total lifetime costs were estimated at €97.4 million (95% CrI: €47.1–158.1 million) for new incident haemophilia cases born in 2011 applying no time discounting, with a mean lifetime cost of €7.8 million per new incident haemophilia case (Table 5). The total lifetime costs, discounted at an annual rate of 3.5%, were estimated at €32.2 million, which is about one third of the undiscounted estimate (Table 5). At almost €92 million, the direct costs accounted for 94.3% of the total costs. Specific haemophilia treatments alone accounted for 77.8% of the total and 82.5% of the direct costs, with €75.4 million for all haemophiliacs (Table 6). Large differences were observed between haemophilia A and B patients regarding treatment costs, with costs for haemophilia A treatment being approximately ten times higher than those for haemophilia B, with haemophilia A incidence being five times higher than haemophilia B incidence. The distributions used in the cost assessment model for the 2011 birth-year Belgian haemophilia patient cohort are shown in Additional files 1 and 2.

**Table 5:** Estimates of 2011 indirect and direct costs due to haemophilia in Belgium

**Scenario**	**No time discounting**		**3.5% time discounting**	
**Cost**	**Mean total cost**	**Mean cost per incident case**	**Mean total cost**	**Mean cost per incident case**
	**(95% CrI) (€)**	**(95% CrI) (€)**	**(95% CrI) (€)**	**(95% CrI) (€)**
**Total**	97,336,761	7,826,097	32,201,550	2,598,176
	(47,139,079–158,080,868)	(3,770,640–12,700,923)	(15,663,972–51,743,686)	(1,242,147–4,192,128)
**Direct**	91,773,744	7,379,337	30,491,342	2,460,299
	(44,416,357–148,640,854)	(3,534,774–11,959,182)	(14,846,997–48,928,191)	(1,173,455–3,955,981)
**Indirect**	5,563,016	446,760	1,710,208	137,878
	(1,657,623–12,092,403)	(134,444–976,977)	(544,059–3,592,225)	(43,083–293,984)

**Table 6:** Estimated costs for haemophilia in Belgium in 2011, by category (in €)

**Categories**	**Mean cost (€)**	**95% credible interval (€)**
Haemophilia treatment	75,446,373	37,224,222–120,744,110
Haemophilia A	69,021,625	33,046,471–110,694,830
Haemophilia B	6,424,748	2,443,720–12,437,337
GP visits	568,828	145,241–1,430,049
Consultation	214,643	81,459–424,020
Transport	354,184	14,568–1,068,605
Specialist visits	1,080,910	269,546–2,632,399
Consultation	441,227	152,963–904,770
Transport	639,684	27,504–1,830,506
Physiotherapist visits	2,024,912	316,369–5,751,834
Consultation	602,380	146,291–1,408,041
Transport	1,422,532	54,573–4,543,715
Dentist visits	320,600	80,680–767,126
Consultation	183,829	52,893–404,829
Transport	136,772	5,148–419,608
Hospitalization	4,906,434	1,305,731–11,200,331
Hospitalization	3,651,264	957,927–8,314,810
Transport	475,853	19,602–1,450,721
Work absence	779,316	211,534–1,755,167
Day hospitalization	4,395,306	707,454–12,810,868
Hospitalization	2,650,648	425,488–7,671,586
Transport	663,505	21,321–2,375,286
Work absence	1,081,153	183,381–3,046,125
Other costs	8,583,380	1,262,101–24,593,803
Unemployment	10,018	2,822–23,224
Invalidity	4,934	1,869–9,560
Death	5,083	0–14,944

GP: General Practitioner.

The cost of health care professional consultations (for GP, specialists, physiotherapists and dentists) represented only 1.6% of total direct costs, with physiotherapist-consultation costs accounting for 41.8% of the total health care visits costs (Table 6). Costs of specialist consultations accounted for 30.6% of these health care visits costs, which was followed by costs related to GP visits, with dentist visits coming last.

The total direct costs for hospital stays and day hospitalisations represented 6.9% of the direct costs. These hospitalisations made up the majority of total indirect costs (53.9%), due to transport and absence-from-work costs.

Sensitivity analyses for the cost assessment indicated that the uncertainty in the total cost estimates was mostly influenced by the uncertainty in incidence rate of haemophilia and other National Institute for Health Care and Disability Insurance (INAMI) costs for patients over 20 years of age (Figure 1B).

## Discussion

DALY calculations for haemophilia in Belgium indicated 145 years lost due to disability or premature death, with a mean of 11 DALYs per incident case. The years lived with disability (YLDs) were seen to have the highest contribution to haemophilia-related DALYs, namely 83.4%, while the years of life lost due to premature mortality (YLLs) were responsible for only 16.6%. This result was not entirely unexpected, as haemophiliac life expectancy closely resembles that of the general population, on account of safe replacement therapies, with the exception of severe haemophiliacs. Moreover, this observation highlights haemophiliac patients’ poorer quality of life compared to that of the general population, although the gap is now closing between the two. In addition, severe haemophiliacs contributed to approximately two thirds of the total DALYs, underlining how far we still have to go to improve severe haemophiliac’s quality-of-life and premature mortality. However, as is a common assumption for all rare diseases, the low total number of haemophilia-related DALYs could lead to haemophilia being viewed as a disease with little burden [32].

To our knowledge, our study was the first of its kind to be conducted in either Belgium or the world. The only published study evaluating evaluate the burden of haemophilia through DALYs involved 19,920 U.S. haemophiliac males, with a total of 107,346 DALYs reported applying 3% time discounting and age weighting [33]. YLL accounted for 12.1% of these DALYs, and YLD for 87.8%. In our study, applying 3% time discounting and age weighting to DALY calculations, YLL accounted for 5.5% of these DALYs, and YLD for 94.5%.

Cost analyses stressed the economic burden due to haemophilia, with total lifetime costs of €97.3 million (95% CrI: €47.1–158.1 million) for new incident 2011-born haemophiliac cases, with a mean lifetime cost of €7.8 million per incident case. Direct costs accounted for 94.3% of the total costs, namely €91.8 million, with a high contribution of treatment-related costs (82.5%) to direct costs and a relatively low contribution of hospitalisation-related costs (6.8%) to direct costs. Large differences in treatment costs were observed between the two types of haemophilia, haemophilia B being five times less frequent than haemophilia A and the proportion of severe haemophilia A patients being higher than the proportion of severe haemophilia B patients in Belgium [1]. The annual cost of specific haemophilia medication injection in 2011 was €65.6 million for clotting factor VIII (ATC code B02BD02), €0.6 million for clotting factor IX (ATC code B02BD04), €6.6 million for nonacog alpha (BeneFIX) (ATC code B02BD09), €1.5 million for factor VIII inhibitor bypassing activity (FEIBA) (ATC code B02BD03), and €6.7 million for activated eptacog alpha (NovoSeven) (ATC code B02BD08). This demonstrates the higher use of replacement therapy for haemophilia A than haemophilia B. However, our study’s total costs are not comparable with those of other studies on account of the incidence approach we used, as well as differences in health care systems (e.g., access to health insurance) and haemophilic medication unit prices between countries. Yet several studies evaluating haemophilia direct costs revealed approximately 85% of direct costs related to anti-haemophilic medication [34-36], in line with the 82.5% found in our study.

A major limitation of our study was the lack of reliable local disease data, as the exact number of haemophiliacs and the distribution of severity types across Belgium were not known and thus had to be estimated. This uncertainty was reflected in the sensitivity analyses that indicated that haemophilia incidence was a major parameter contributing to the overall uncertainty. Furthermore, as there was only scarce information on the number of patients developing inhibitors, we were not able to perform precise health and economic burden assessments for this patient population. The total lifetime costs could be very different for patients with inhibitors compared to those without. On the other hand, it must be mentioned that cost estimates were calculated using a representative Belgian database from the largest national sickness fund. Lastly, we conducted uncertainty analyses, propagating the uncertainty in the input parameters to the final DALY or cost estimate.

In this study, the future health and economic burden of haemophilia was predicted using the incidence approach. To this end, we assumed that haemophilia incidence would remain constant over time, which we consider justified for this rare genetic disease. Yet the future burden of haemophilia is likely to change with the development of innovative treatments, such as long-acting clotting agents or gene therapy, or the availability of Biosimilars [1]. In addition, the introduction of these treatments will likely impact future costs related to haemophilia.

The extensive use of effective prophylactic clotting factor replacement therapies, especially in children and severe haemophiliacs, has considerably pushed up treatment-related costs in Belgium, with a total cost of 33 million euro in 2002 up to 81 million euro in 2011 [37]. Although the use of an effective prophylactic approach may thus seem costly on the surface, several studies have shown that prophylaxis minimises and prevents long-term haemophilia complications, such as arthropathies, as well as increased disability and earlier death. Moreover, early introduction of prophylaxis may help to reduce the cost of inhibitor-related costs [38]. Consequently, prophylaxis appears crucial improving the health and economic consequences of haemophilia, ensuring a better quality of life for haemophiliacs and hence a lower disease burden [1,39,40]. Given the current economic downturn, individualised therapy, appropriate pharmacokinetic-dosing and/or tender processes may help to reduce costs [41,42]. For recombinant products similar prices have been maintained since 1991 and the revision of this issue might help to support the treatment of patients at a lower cost. In addition, increasing expensive rare disease therapies could stimulate competition, potentially leading to price competitiveness of such therapies. It should also be noted that FVIII and FIX concentrates are considered essential medicines by the World Health Organisation (WHO) for both adults and children according to the 17^th^ WHO Essential Medicines List and the 3^rd^ WHO Essential Medicines List for Children [43].

In conclusion, although haemophilia has a minor impact on the overall disease burden, the repercussions it has on the individual patient remain substantial. Initiatives for reducing this burden should be clearly identified and given full support. To reduce haemophilia treatment costs, a compromise must be made between the patient benefitting from the treatment, for whom the treatment has to be available and affordable, and the national authorities facing budget constraints, as well as the pharmaceutical companies investing in research and development [44]. Finally, research and development of new drugs in the haemophilia field still needs to be further promoted.

## Abbreviations

CF: Clotting factor; CrI: Credible interval; DALY: Disability-adjusted life year; DW: Disability weight; FVIII: Factor VIII; FIX: Factor IX; GBD: Global burden of disease; GP: General practitioner; HA: Haemophilia A; HB: Haemophilia B; KCE: Belgian health care knowledge centre; NACM: National alliance of christian mutualities; NIHCDI: National institute for health care and disability insurance (INAMI/RIZIV); PWH: Person with haemophilia; WHO: World health organization; YLD: Years lived with disability; YLL: Years of life lost.

## Competing interests

Michael Callens and Frank De Smet are employed by the National Alliance of Christian Sickness Funds, a large Belgian sickness fund (health care insurer).

Séverine Henrard, Brecht Devleesschauwer, Philippe Beutels, Cedric Hermans and Niko Speybroeck have no competing interests.

## Authors’ contributions

SH and BD were involved in design, acquisition and analysis of data, drafted the manuscript and revised critically the manuscript. NS and CH were involved in design and acquisition of data, and revised critically the manuscript. PB was involved in design of the study and revised the manuscript. FS and MC were involved in acquisition of data and revised the manuscript. All authors have read and approved the final manuscript.

## Supplementary Material

Additional file 1: Table S1 Distributions used in the cost assessment model for the 2011 birth-year Belgian haemophilia cohort. Click here for file

Additional file 2: Table S2 Distributions of costs (in euros) in the cost assessment model used for the 2011 birth-year Belgian haemophilia cohort. Click here for file
