# Supplementary material for: The health and economic burden of haemophilia in Belgium: a rare, expensive and challenging disease
Source: Orphanet J Rare Dis. 2014 Mar 21;9:39. doi: 10.1186/1750-1172-9-39 (PMC3998193; doi:10.1186/1750-1172-9-39)
Supplement: Additional file 1: Table S1 — Distributions used in the cost assessment model for the 2011 birth-year Belgian haemophilia cohort. [file 1750-1172-9-39-S1.pdf]

Additional file

Format:PDF

File name: Additional_file_1.pdf

Additional file 1: Table S1. Distributions used in the cost assessment model for the 2011 birth-year Belgian haemophilia cohort

Parameter	Distribution	Reference
Annual number of home visits from a general practitioner during the day		
Annual number of home visits of a general practitioner during the day for patients aged 0-10	Beta-PERT(min = 0, mode = 0, max = 6)	NACM**
Annual number of home visits of a general practitioner during the day for patients aged 10-20	Beta-PERT(min = 0, mode = 0, max = 3)	NACM**
Annual number of home visits of a general practitioner during the day for patients aged 20-40	Beta-PERT(min = 0, mode = 0, max = 4)	NACM**
Annual number of home visits of a general practitioner during the day for patients aged 40-60	Beta-PERT(min = 0, mode = 0, max = 10)	NACM**
Annual number of home visits of a general practitioner during the day for patients aged 60+	Beta-PERT(min = 0, mode = 0, max = 26)	NACM**
Annual number of home visits from a general practitioner during the night or weekend		
Annual number of home visits of a general	Beta-PERT(min = 0, mode = 0, max = 1)	NACM**

practitioner during the night or weekend for patients aged 0-10		
Annual number of home visits of a general practitioner during the night or weekend for patients aged 10-20	Beta-PERT(min = 0, mode = 0, max = 1)	NACM**
Annual number of home visits a general practitioner during the night or weekend for patients aged 20-40	Beta-PERT(min = 0, mode = 0, max = 1)	NACM**
Annual number of home visits of a general practitioner during the night or weekend for patients aged 40-60	Beta-PERT(min = 0, mode = 0, max = 1)	NACM**
Annual number of home visits of a general practitioner during the night or weekend for patients aged 60+	Beta-PERT(min = 0, mode = 0, max = 7)	NACM**
Annual number of practice visits to a general practitioner during the day		
Annual number of practice visits to a general practitioner during the day for patients aged 0-10	Beta-PERT(min = 0, mode = 0, max = 11)	NACM**
Annual number of practice visits to a general practitioner during the day for patients aged 10-20	Beta-PERT(min = 0, mode = 0, max = 21)	NACM**
Annual number of practice visits to a general practitioner during the day for patients aged 20-40	Beta-PERT(min = 0, mode = 0, max = 23)	NACM**
Annual number of practice visits to a general practitioner during the day for patients aged 40-60	Beta-PERT(min = 0, mode = 0, max = 33)	NACM**

Annual number of practice visits to a general practitioner during the day for patients aged 60+	Beta-PERT(min = 0, mode = 0, max = 7)	NACM**
Annual number of practice visits to a general practitioner during the night or weekend		
Annual number of practice visits to a general practitioner during the night or weekend for patients aged 0-10	Beta-PERT(min = 0, mode = 0, max = 4)	NACM**
Annual number of practice visits to a general practitioner during the night or weekend for patients aged 10-20	Beta-PERT(min = 0, mode = 0, max = 17)	NACM**
Annual number of practice visits to a general practitioner during the night or weekend for patients aged 20-40	Beta-PERT(min = 0, mode = 0, max = 3)	NACM**
Annual number of practice visits to a general practitioner during the night or weekend for patients aged 40-60	Beta-PERT(min = 0, mode = 0, max = 34)	NACM**
Annual number of practice visits to a general practitioner during the night or weekend for patients aged 60+	Beta-PERT(min = 0, mode = 0, max = 4)	NACM**
Annual number of practice visits to a specialist during the day		
Annual number of practice visits to a specialist during the day for patients aged 0-10	Beta-PERT(min = 0, mode = 2, max = 116)	NACM**
Annual number of practice visits to a specialist during the day for patients aged 10-20	Beta-PERT(min = 0, mode = 6, max = 63)	NACM**
Annual number of practice visits to a specialist during the day for patients aged 20-40	Beta-PERT(min = 0, mode = 0, max = 30)	NACM**
Annual number of practice visits to a specialist during the day for patients aged 40-60	Beta-PERT(min = 0, mode = 2, max = 36)	NACM**

Annual number of practice visits to a specialist during the day for patients aged 60+	Beta-PERT(min = 0, mode = 5, max = 26)	NACM**
Annual number of practice visits to a specialist during the night or weekend		
Annual number of practice visits to a specialist during the night or weekend for patients aged 0-10	Beta-PERT(min = 0, mode = 0, max = 14)	NACM**
Annual number of practice visits to a specialist during the night or weekend for patients aged 10-20	Beta-PERT(min = 0, mode = 0, max = 6)	NACM**
Annual number of practice visits to a specialist during the night or weekend for patients aged 20-40	Beta-PERT(min = 0, mode = 0, max = 2)	NACM**
Annual number of practice visits to a specialist during the night or weekend for patients aged 40-60	Beta-PERT(min = 0, mode = 0, max = 0)	NACM**
Annual number of practice visits to a specialist during the night or weekend for patients aged 60+	Beta-PERT(min = 0, mode = 0, max = 0)	NACM**
Annual number of practice visits to a physiotherapist		
Annual number of practice visits to a physiotherapist for patients aged 0-10	Beta-PERT(min = 0, mode = 0, max = 13)	NACM**
Annual number of practice visits to a physiotherapist for patients aged 10-20	Beta-PERT(min = 0, mode = 0, max = 115)	NACM**
Annual number of practice visits to a physiotherapist for patients aged 20-40	Beta-PERT(min = 0, mode = 0, max = 82)	NACM**
Annual number of practice visits to a physiotherapist for patients aged 40-60	Beta-PERT(min = 0, mode = 0, max = 206)	NACM**

Annual number of practice visits to a physiotherapist for patients aged 60+	Beta-PERT(min = 0, mode = 0, max = 185)	NACM**
Annual number of practice visits to a dentist		
Annual number of practice visits to a dentist for patients aged 0-10	Beta-PERT(min = 0, mode = 0, max = 6)	NACM**
Annual number of practice visits to a dentist for patients aged 10-20	Beta-PERT(min = 0, mode = 1, max = 18)	NACM**
Annual number of practice visits to a dentist for patients aged 20-40	Beta-PERT(min = 0, mode = 0, max = 16)	NACM**
Annual number of practice visits to a dentist for patients aged 40-60	Beta-PERT(min = 0, mode = 0, max = 7)	NACM**
Annual number of practice visits to a dentist for patients aged 60+	Beta-PERT(min = 0, mode = 0, max = 14)	NACM**
Annual number of hospitalisation days		
Annual number of hospitalisation days for patients aged 0-10	Beta-PERT(min = 0, mode = 0, max = 37)	NACM**
Annual number of hospitalisation days for patients aged 10-20	Beta-PERT(min = 0, mode = 0, max = 43)	NACM**
Annual number of hospitalisation days for patients aged 20-40	Beta-PERT(min = 0, mode = 0, max = 30)	NACM**
Annual number of hospitalisation days for patients aged 40-60	Beta-PERT(min = 0, mode = 0, max = 56)	NACM**
Annual number of hospitalisation days for patients aged 60+	Beta-PERT(min = 0, mode = 0, max = 51)	NACM**
Annual number of day hospitalisations		
Annual number of day hospitalisations for	Beta-PERT(min = 0, mode = 0, max = 79)	NACM**

patients aged 0-10		
Annual number of day hospitalisations for patients aged 10-20	Beta-PERT(min = 0, mode = 0, max = 17)	NACM**
Annual number of day hospitalisations for patients aged 20-40	Beta-PERT(min = 0, mode = 0, max = 20)	NACM**
Annual number of day hospitalisations for patients aged 40-60	Beta-PERT(min = 0, mode = 0, max = 172)	NACM**
Annual number of day hospitalisations for patients aged 60+	Beta-PERT(min = 0, mode = 0, max = 5)	NACM**
Average distance to a hospital or practitioner (km)	Uniform(0, 200)	Estimation
Probability of developing invalidity	Beta(24, 771)	NIHCDI*
Friction period	Uniform(60, 180)	KCE [30]

* NIHCDI = National institute for health care and disability insurance (INAMI/RIZIV); ** NACM = National alliance of Christian mutualities; KCE = Belgian health care knowledge centre
