# Supplementary material for: The health and economic burden of haemophilia in Belgium: a rare, expensive and challenging disease
Source: Orphanet J Rare Dis. 2014 Mar 21;9:39. doi: 10.1186/1750-1172-9-39 (PMC3998193; doi:10.1186/1750-1172-9-39)
Supplement: Additional file 2: Table S2 — Distributions of costs (in euros) in the cost assessment model used for the 2011 birth-year Belgian haemophilia cohort. [file 1750-1172-9-39-S2.pdf]

Additional file

Format: PDF

File name: Additional_file_2.pdf

Additional file 2: Table S2. Distributions of costs (in euros) in the cost assessment model used for the 2011 birth-year Belgian haemophilia cohort

**Parameter**	**Distribution**	**Reference**
Haemophilia treatment costs		
Annual treatment costs for HA patients	Gamma(shape = 73842426, rate = 941)	NIHCDI*
Annual treatment costs for HB patients	Gamma(shape = 7259986, rate = 212)	NIHCDI*
Medical and paramedical consultation costs		
Average cost of a general practitioner consultation for the NIHCDI*	21.40	NACM**
Average cost of a general practitioner consultation for the patient	4.55	NACM**
Average cost of a specialist consultation for the NIHCDI*	18.29	NACM**
Average cost of a specialist consultation for the patient	23.24	NACM**
Average cost of a physiotherapist consultation for the NIHCDI*	17.64	NACM**
Average cost of a physiotherapist	7.89	NACM**

consultation for the patient		
Average cost of a dentist consultation for the NIHCDI*	49.48	NACM**
Average cost of a dentist consultation for the patient (age 0-20)	79.09	NACM**
Average cost of a dentist consultation for the patient (age 20+)	12.96	NACM**
Hospitalisation costs		
Average hospitalisation cost for the NIHCDI* (age 0-20), per day	341.10	NACM**
Average hospitalisation cost for the NIHCDI* (age 20+), per day	410.10	NACM**
Average hospitalisation cost for the patient (age 0-20), per day	25.38	NACM**
Average hospitalisation cost for the patient (age 20+), per day	80.84	NACM**
Day hospitalisation costs		
Average day hospitalisation cost for the NIHCDI* (age 0-20)	179.40	NACM**
Average day hospitalisation cost for the NIHCDI* (age 20+)	224.10	NACM**
Average day hospitalisation cost for the patient (age 0-20)	13.15	NACM**
Average day hospitalisation cost for	29.78	NACM**

the patient (age 20+)		
Other costs		
Average other costs for the NIHCDI* (age 0-20)	Beta-PERT(min = 10.04, mode = 435.20, max = 30890.00)	NACM**
Average other costs for the NIHCDI* (age 20+)	Beta-PERT(min = 0.00, mode = 445.20, max = 47460.00)	NACM**
Average other costs for the patient (age 0-20)	Beta-PERT(min = 1.75, mode = 63.28, max = 728.50)	NACM**
Average other costs for the patient (age 20+)	Beta-PERT(min = 0.00, mode = 88.85, max = 3251.00)	NACM**
Average salary	3000	Estimation
Average cost per kilometre travelled	0.30	Estimation

* NIHCDI = National institute for health care and disability insurance (INAMI/RIZIV); ** NACM = National alliance of Christian mutualities
